# Investigating seasonal changes in factors associated with COVID-19 concerns: Results from a serial cross-sectional survey study in Germany between 2020 and 2023

**DOI:** 10.3389/fpubh.2024.1397283

**Published:** 2024-07-18

**Authors:** Robin Janzik, Dan Borzekowski, Gaby-Fleur Böl

**Affiliations:** Department Risk Communication, German Federal Institute for Risk Assessment (BfR), Berlin, Germany

**Keywords:** COVID-19, concerns, behavior, perceptions, serial cross-sectional data, survey study

## Abstract

**Objective:**

COVID-19 risk perceptions are discussed to be volatile and have been shown to be connected to the adoption of preventive public health behaviors. This study aimed to investigate changes in COVID-19 concerns and influencing factors as a function of season among the German public.

**Methods:**

Sixty-three waves of cross-sectional telephone surveys with German participants aged 14 years and older conducted at least monthly between June 2020 and April 2023 provided the data basis (*N* = 63,471). After pooling participants of different waves by season (spring, summer, fall, winter), data were analyzed with regard to changes in physical health, mental health, economic, and social COVID-19 concerns. Individual characteristics (e.g., age), COVID-19 behavior (e.g., hygiene practices), and related perceptions (e.g., controllability of risk) were considered as predictors of composite concerns in different seasons.

**Results:**

Results showed a higher between-seasons than within-seasons variability in concerns, with rises in physical and mental health and social concerns during fall. Multivariate regressions revealed being female, lower education, adopting protective measures, and higher perceived probability of infection in both public and private settings to be consistent predictors of higher COVID-19 concerns. Coefficients of these predictors remained comparatively stable over seasons and years.

**Conclusion:**

Results indicate re-occurring changes in concerns during a prolonged crisis, with distinct characteristics being consistently associated with higher reported concerns. To ensure the application of protective measures, communicators should consider that risk perceptions are subject to fluctuations, but that certain groups of individuals tend to develop them and therefore deserve particular focus.

## 1 Introduction

### 1.1 Prolonged public health crises and the development of concerns

The global COVID-19 pandemic has had a profound impact on people’s lives in many ways in recent years. Not only have people been physically affected by the disease itself caused by SARS-CoV-2, but there have also been problems with mental health as a result of measures taken by governments (1, 2). Other affected areas relate to financial conditions, for example due to the loss of jobs (3), or social issues due to fewer and altered opportunities for interpersonal interaction (4). Individuals form their own opinions on these topics and such perceptions can have an impact on behavior with regard to compliance with measures (5). In this context, several studies have shown that risk perceptions in relation to COVID-19 were associated with general behavior change (6) or adopting specific protective measures (7).

Accordingly, understanding risk perceptions has been an important task of research during the pandemic. To investigate risk perceptions, studies often measure individuals’ concerns about a topic (8). The literature provides several approaches to conceptualize concerns, with contemporary approaches focusing on cognitive processes and viewing concerns as a product of thought patterns (9). Relatedly, concerns can develop through an interplay of different cognitive, emotional, and social factors (10), stressing the importance of context and culture (11). A number of studies provided evidence of high levels of concerns about COVID-19 in many regions of the world (12).

Further research has examined which individual and contextual factors influence these perceptions in different countries. For instance, studies have explored the impact of sociodemographic characteristics such as gender, age, and education on concerns. While some studies found higher concerns in female participants (13, 14), others did not find an effect of gender (15). In terms of age, some works showed positive associations with levels of concerns (16) and others negative associations (17). A more conclusive picture emerged for effects of education, as several studies found less educated participants to be more concerned (3, 18). Another important set of variables to explain concerns have been related perceptions, as these are assumed to provide the basis for the development of concerns. For instance, Solymosi et al. (19) found lower perceived control in participants exhibiting higher levels of worry about COVID-19, while Khaira and Sari (4) showed perceived susceptibility to being infected to be positively associated with worry. Further studies provided evidence of negative associations of knowledge about COVID-19 (20) and perceived appropriateness of government measures (21) with concerns. Similarly, some studies investigated the relationship between COVID-19-related behavior and concerns. Participants adopting preventive measures (e.g., social isolation) were more likely to report higher levels of concerns (22).

However, the majority of these existing studies focused either on individual points in time during the pandemic or on relatively short periods. As the development of concerns about a specific object can be subject to changes over time due to adjustment to circumstances (23, 24), studies over longer periods could help to better understand the potential variability of concerns and identify relevant influencing factors. What the literature is lacking so far is the perspective on seasonality in this context. As with other respiratory diseases, seasonal fluctuations in the spread of COVID-19 have been observed in temperate regions (25–27). While it is assumed that climatic conditions such as temperature and humidity affect the survival of the virus, mechanisms of transmission are less clear (28). This much-discussed effect of season could also play a role in shaping conditions for the development of concerns. Transmission patterns in the form of peaks in infection numbers in fall and winter were directly observable for the public, and governments as well as health organizations often intensified communication efforts in colder months (29, 30). Moreover, behavioral changes, such as spending more time indoors during colder months (31), and seasonal variations in media coverage, with potentially increased attention during infection outbreaks, could contribute to increased awareness, potentially influencing risk perceptions.

Against this background, further investigations into the research gaps on the development of concerns about COVID-19 over longer periods during the pandemic, on the effect of season, and on the consistent nature of influencing factors of concerns appear to be useful.

### 1.2 The current study

This study aimed to add a temporal perspective to the literature on risk perceptions of COVID-19, with a particular focus on the effect of season. The examination of concerns provides insights into a core variable in the process of health behavior in the context of infectious diseases (5, 12). The study draws on data from Germany that span several years and cover various aspects of how individuals deal with COVID-19, reflecting important determinants of health-related conditions as assessed in epidemiological studies. This allows a comprehensive view of the pandemic and thus goes beyond the consideration of individual key events (e.g., the introduction of vaccinations). The deeper understanding of the timely course and correlates of concerns helps to determine which individuals show concerns at which times and are therefore more likely to adapt their behavior. These findings offer starting points for risk communication in the light of future health crises and can also aid clinical decision-making by understanding prospective patients’ ways of thinking. Addressing the research gaps outlined above, the study was guided by these research questions:

RQ1: How do concerns about COVID-19 vary with different seasons?

RQ2: How do concerns about COVID-19 in different seasons relate to (a) individual characteristics, (b) related behavior, and (c) related perceptions?

## 2 Methods

### 2.1 Procedure and participants

The current study used data from the BfR-Corona-Monitor (32).^1^ This multi-wave, cross-sectional telephone survey of the general population in Germany aged 14 and over was conducted between 2020 and 2023 in cooperation with a professional service provider for market and social research. The participants answered the questions as part of an omnibus survey (i.e., together with other topics that changed per wave). This procedure reduces the effect of self-selection, as participants cannot make their participation dependent on their interest in a particular topic.

The data used here comprised a total of 63 of 73 waves of the survey, which were conducted at least monthly between June 2020 and April 2023, each on two consecutive days. Sample sizes ranged between 978 and 1,037 respondents per wave. Data included a total of *N* = 63,471 participants. Based on sociodemographic characteristics, the sample resembled the German population (see Table 1).

**Table 1:** Participants’ overall sociodemographic characteristics.

		*n*	%
Gender	Male	30,526	48.1
	Female	32,945	51.9
Age	*M*, *SD*	57.5	17.5
	14–17 years	1,222	1.9
	18–29 years	4,380	6.9
	30–39 years	4,762	7.5
	40–49 years	7,562	11.9
	50–59 years	13,806	21.8
	60–69 years	15,123	23.8
	70–79 years	10,063	15.9	80 years and older	6,553	10.3
Education	Lower	11,150	17.6
	Medium	18,175	28.6
	Higher	34,146	53.8
Occupation	Non-full-time	33,110	52.2
	Full-time	30,361	47.8
Household size	*M*, *SD*	2.2	1.1

*N* = 63,471.

To compare seasons, participants of each wave were pooled based on the month data collection took place in: summer (June, July, August), fall (September, October, November), winter (December, January, February), spring (March, April, May); this was done for each year, respectively, resulting in 12 segments (see Supplementary material).

### 2.2 Measurements

#### 2.2.1 COVID-19 concerns

The measurement of COVID-19 concerns included four items beginning with the question “To what extent are you personally concerned or not concerned about the impact of the novel coronavirus in the following areas of life?” Reflecting central, overarching domains, these were: economic situation, social relationships, physical as well as mental health. Items were measured on a 5-point scale ranging from 1 = “not concerned at all” to 5 = “very concerned.” The mean index showed satisfying reliability across waves (α_Range_ = 0.62–0.74).

#### 2.2.2 Individual characteristics

The measurement of individual characteristics comprised five socio-demographics. *Gender* was measured dichotomously (1 = male, 2 = female) and *age* continuously in years. The measurement of *education* was based on five increasing levels from 1 = “pupil” to 5 = “academic degree (university, academy, polytechnic).” The dichotomous measurement of *occupation* compared participants in terms of their employment status (1 = non-full-time, 2 = full-time) and with *household size*, the number of people living in each participant’s home was assessed.

#### 2.2.3 COVID-19 behavior

COVID-19 behavior was measured retrospectively using 11 items following the multiple-selection question “Which of the following measures have you taken within the past 2 weeks to protect yourself or others from the novel coronavirus?” Based on theoretical argument, items were pooled across waves into the three constructs *hygiene* (4 items; e.g., “washed hands more thoroughly”), *isolation* (3 items; e.g., “met friends or family less frequently”), and *provision* (4 items; e.g., “built up larger stocks”). Reliability scores were below common thresholds; however, this was not deemed problematic given the formative measurement model. The inclusion began in the segment for summer 2021.

#### 2.2.4 COVID-19 perceptions

The measurement of COVID-19 perceptions included seven constructs. *Controllability of risk* measured participants’ level of certainty regarding protection from infection (1 item; 5-point scale from 1 = “not sure at all” to 5 = “very sure”). Participants evaluated the appropriateness of two COVID-19 measures, the *mask mandate* and the *cancelation of events* (each 1 item, 2-point scale from 1 = “not appropriate” to 2 = “appropriate”). Participants’ perceived level of information was measured by asking them how well they feel informed about central COVID-19 topics (5-point scale from 1 = “not well informed at all” to 5 = “very well informed”); after pooling corresponding items, the two indices *virus* (3 items; e.g., “infection routes of the coronavirus”) and *societal implications* (4 items; e.g., “measures currently in effect”) were built. Finally, participants estimated the probability of infection for different locations on a 5-point scale ranging from 1 = “very low” to 5 = “very high”; corresponding items were pooled within the two indices *private context* (2 items; e.g., “at home”) and *public context* (5 items; e.g., “workplace”). The inclusion of measurements regarding level of information and probability of infection began in the segment for summer 2022.

Table 2 gives an overview of descriptive statistics for the measurements related to COVID-19.

**Table 2:** Descriptive statistics and internal consistency of measured variables.

	Items	*n*	Range	*M*	*SD*	α_Total_	α_Range_
1. COVID-19 concerns	4	63,424	1–5	2.30	0.91	0.68	0.62–0.74
COVID-19 behavior							
2. Protective measures – hygiene	4	42,139	0–1	0.64	0.29	0.52	0.43–0.59
3. Protective measures – isolation	3	42,139	0–1	0.51	0.37	0.64	0.57–0.60
4. Protective measures – provision	4	42,139	0–1	0.29	0.22	0.27	0.20–0.36
COVID-19 perceptions							
5. Controllability of risk	1	62,408	1–5	3.26	1.23	–	–
6. Appropriateness – masks	1	63,016	1–2	1.10	0.31	–	–
7. Appropriateness – events	1	61,600	1–2	1.22	0.41	–	–
8. Feeling informed – virus	3	19,881	1–5	3.68	1.00	0.72	0.68–0.73
9. Feeling informed – society	4	19,900	1–5	3.59	0.97	0.70	0.69–0.71
10. Infection probability – private	2	19,877	1–5	1.54	0.77	–	–
11. Infection probability – public	5	19,881	1–5	3.22	0.88	0.77	0.74–0.83

*n* = 19,877–63,424.

### 2.3 Data analysis

In line with Haddad et al. (35) and similar to Betsch et al. (36), this study used unweighted data. As weighting would be based on both variables included as predictors in regression models (e.g., gender) and variables not considered in them (e.g., federal state), it could not be guaranteed that the relationship between the measured weighting variables and the outcome variable of concerns would be correctly specified, thus potentially introducing additional bias in analyses (37–39). However, respecifying models using weighted data led to similar results.

Analyses regarding RQ1 were based on inspecting means and standard deviations in the 12 different season segments as well as a series of one-way between-subjects ANOVAs to compare the effect of seasons on types of concerns. Considering inequality of variances, Games-Howell post-hoc tests were computed for individual comparisons.

In terms of RQ2 regarding predictors and changes over time, a series of linear multivariate regression analyses predicting the mean index of concerns was performed. All predictors at hand were simultaneously included in the analysis of each of the 12 season segments. Relevant statistical requirements (e.g., multicollinearity) for the analyses were checked in advance. *A-priori* power analyses using G*Power (version 3.1) (40) for small effects (*f*^2^ = 0.02) at α = 0.05 and 1–β = 0.99 indicated required sample sizes of at least 1,526 (8 predictors, summer 2020 to spring 2021), 1,675 (11 predictors, summer 2021 to winter 2021), and 1,842 (15 predictors, spring 2022 to spring 2023). Given pooled sample sizes ranging from 1,844 to 6,789 participants per season segment, analyses were sufficiently powered.

Data preparation was done using *SPSS* (version 26) (41). Analyses were performed in *R* (version 4.3.2) (42).

## 3 Results

### 3.1 Changes in COVID-19 concerns over time (RQ1)

**Figure 1:**
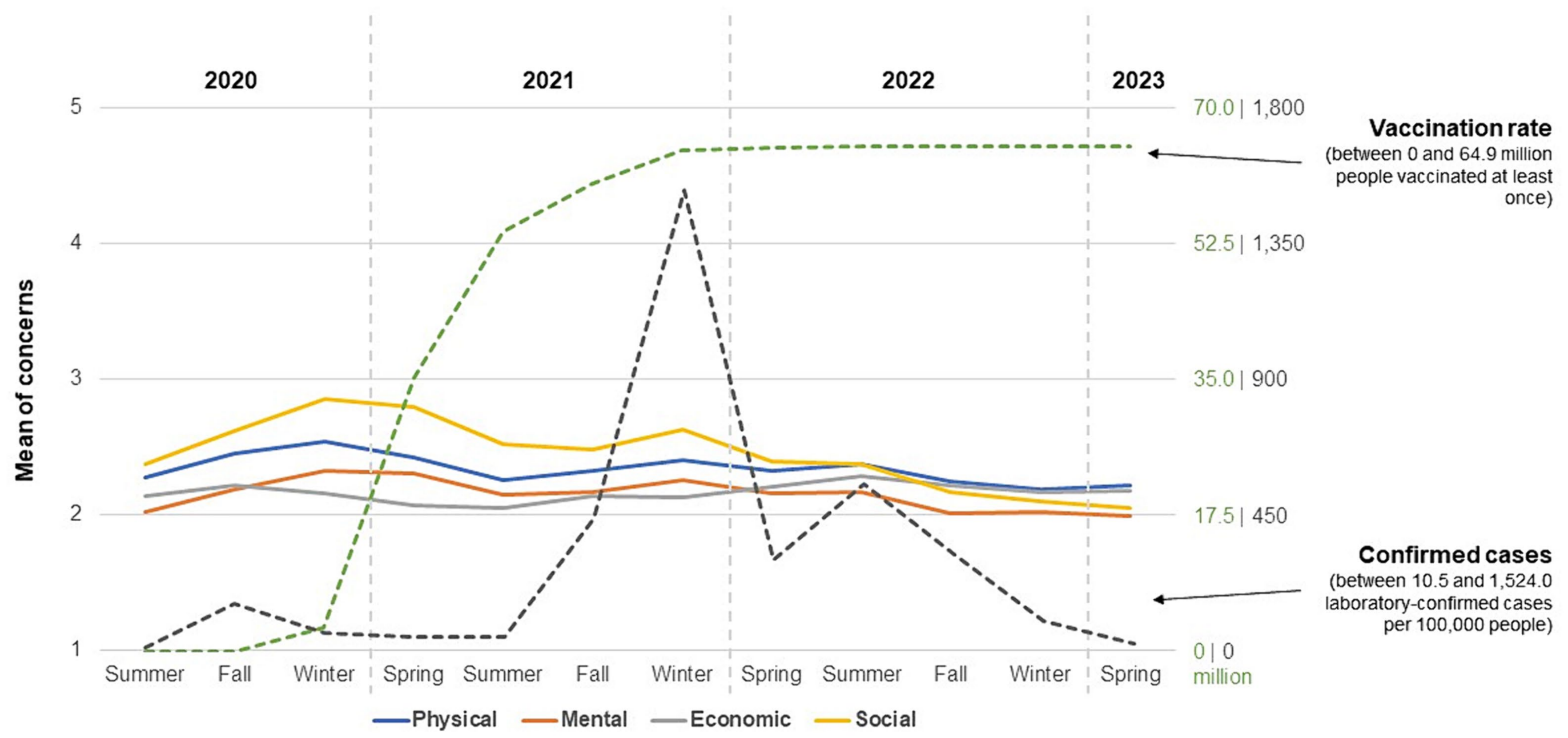
Means of concerns by season in connection to vaccination rate and confirmed cases. *n*_Seasons_ = 1,946–7,095. Vaccination rate in Germany (number of people vaccinated at least once in millions) based on https://impfdashboard.de/. Confirmed cases in Germany (number of laboratory-confirmed COVID-19 cases per 100,000 inhabitants per week) based on https://infektionsradar.gesund.bund.de/de/covid/inzidenz/. Values of vaccination rate and confirmed cases per season based on the last day of each segment’s survey data collection, respectively.

RQ1 investigated the time-dependent course of concerns about the impact of COVID-19 on different areas of participants’ lives and whether there were effects of season. Figure 1 gives an impression of the distribution of the different types of concerns between 2020 and 2023. At first, results showed that respondents’ concerns were in the lower half of the 5-point scale over the entire investigated period. The highest value was found for social concerns in winter 2020 (*M* = 2.85, *SD* = 1.33), while the lowest was measured for mental concerns in spring 2023 (*M* = 1.99, *SD* = 1.23). Over the entire period, social concerns were the most pronounced (*M* = 2.51, *SD* = 1.30), followed by physical health concerns (*M* = 2.36, *SD* = 1.27), with mental (*M* = 2.17, *SD* = 1.24) and economic (*M* = 2.16, *SD* = 1.26) concerns showing lower but similar values. The overall variability of values was similar over time (*SD* = 1.18–1.35).

Descriptive data also suggest differences depending on season. With regard to physical health concerns, average values in spring (*M* = 2.22–2.42) and summer (*M* = 2.26–2.37) were lower than those in fall (*M* = 2.25–2.45) and winter (*M* = 2.19–2.54). Mean values differed significantly by season, *F*(3, 34,951) = 24.25, *p* < 0.001, ω^2^ = 0.002. Post-hoc comparisons indicated that these concerns differed by each season (*p*s < 0.05). However, only the comparison of spring and winter showed no difference (*p* = 0.883).

A slightly different picture emerged for mental health concerns. There were different ranges of mean values for winter (*M* = 2.02–2.33) and spring (*M* = 1.99–2.31) compared to summer (*M* = 2.02–2.17) and fall (*M* = 2.01–2.19). An ANOVA again showed a significant effect of season, *F*(3, 34,895) = 34.79, *p* < 0.001, ω^2^ = 0.003, with post-hoc comparisons indicating differences between each season (*p*s < 0.05) with one exception; there was no difference between spring and fall (*p* = 0.112).

In the case of economic concerns, values were again more comparable in spring (*M* = 2.07–2.21) and summer (*M* = 2.05–2.29) as opposed to fall (*M* = 2.14–2.22) and winter (*M* = 2.13–2.17). Season also had a significant influence on being concerned about economic consequences, *F*(3, 34,846) = 5.29, *p* = 0.001, ω^2^ = 0.000. Post-hoc comparisons showed differences in each case between spring, summer, and fall paired with winter (*p*s < 0.05).

In terms of concerns about the development of social relationships, values for fall (*M* = 2.17–2.62), winter (*M* = 2.10–2.85), and spring (*M* = 2.05–2.80) were similar, while the range of means for summer (*M* = 2.37–2.52) was comparatively smaller. There was also an influence of the season here, *F*(3, 34,771) = 57.99, *p* < 0.001, ω^2^ = 0.005, with *post-hoc* comparisons indicating significant differences between each time segment (*p*s < 0.05).

Finally, results showed season to affect the mean index of concerns, *F*(3, 35,085) = 41.87, *p* < 0.001, ω^2^ = 0.003.

### 3.2 Predictors of COVID-19 concerns over time (RQ2)

RQ2 aimed to examine possible factors associated with COVID-19 concerns depending on season. First inspecting correlations in the whole sample revealed all investigated variables to be significantly correlated with concerns (see Supplementary material).

As can be seen in Table 3 on results of multivariate regressions, significant predictors remained comparatively stable over seasons. In response to RQ2a on individual characteristics, females were more likely to report concerns about the effects of COVID-19 in 10 of 12 time segments (*β* = 0.06–0.12, *p*s < 0.001). While age was a significant negative predictor in winter 2020 as well as spring, summer, and winter 2021 (*β* = −0.04–0.10, *p*s < 0.01), the effect diminished with the inclusion of further perceptions into analyses. Conversely, lower education was significantly associated with higher levels of concerns consistently over all time segments (*β* = −0.07–0.13, *p*s < 0.001). With exceptions of smaller effects in summer and winter of 2022 (*β* = 0.05, *p*s < 0.05), being full-time worker did not significantly predict concerns. Regarding the influence of household size, there was no obvious pattern. Having fewer people in the household was associated with stating higher concerns in summer and fall 2020, spring and summer 2021, as well as spring 2022 (*β* = −0.03–0.05, *p*s < 0.05), but there were no effects in the seven remaining time segments.

**Table 3:** Results of regression analyses predicting COVID-19 concerns by season.

	2020			2021				2022				2023	Overall
	Summer	Fall	Winter	Spring	Summer	Fall	Winter	Spring	Summer	Fall	Winter	Spring	
1. Gender	0.08***	0.08***	0.10***	0.12***	0.10***	0.08***	0.09***	0.09***	0.07***	0.02	0.06***	0.03	0.07***
2. Age	0.00	−0.01	−0.07***	−0.10***	−0.07***	−0.01	−0.04**	−0.02	−0.00	−0.04	−0.02	0.04	−0.01
3. Education	−0.12***	−0.12***	−0.09***	−0.08***	−0.09***	−0.08***	−0.10***	−0.08***	−0.09***	−0.07***	−0.13***	−0.11***	−0.09***
4. Occupation	0.01	0.01	−0.02	−0.00	0.02	0.02	0.00	−0.01	0.05*	0.02	0.05*	−01	0.01
5. Household size	−0.04*	−0.04**	−0.02	−0.03*	−0.05***	−0.01	−0.02	−0.04**	−0.03	−0.03	−0.04	−0.04	−0.04***
6. Protective measures – hygiene	–	–	–	–	0.05**	0.03**	0.03*	0.04**	0.05*	0.06**	0.08***	0.05	0.05***
7. Protective measures – isolation	–	–	–	–	0.12***	0.21***	0.18***	0.14***	0.13***	0.20***	0.14***	0.08**	0.16***
8. Protective measures – provision	–	–	–	–	0.04**	0.04**	0.04**	0.04**	0.01	0.02	0.03	0.02	0.03***
9. Controllability of risk	−0.14***	−0.13***	−0.12***	−0.14***	−0.10***	−0.12***	−0.08***	−0.05***	−0.04*	−0.03	−0.03	−0.00	−0.04***
10. Appropriateness – masks	−0.03*	−0.03**	0.02	0.03*	0.03*	0.05***	0.05***	0.05***	0.05*	0.02	0.04*	0.09***	0.05***
11. Appropriateness – events	0.00	−0.00	0.03	0.05***	0.01	−0.01	−0.01	−0.00	−0.01	−0.03	0.01	−0.02	−0.01
12. Feeling informed – virus	–	–	–	–	–	–	–	−0.06***	−0.06*	−0.09***	−0.01	−0.08*	−0.06***
13. Feeling informed – society	–	–	–	–	–	–	–	−0.06***	−0.03	−0.04	−0.07**	0.03	−0.04***
14. Infection probability – private	–	–	–	–	–	–	–	0.09***	0.14***	0.10***	0.13***	0.10***	0.11***
15. Infection probability – public	–	–	–	–	–	–	–	0.11***	0.15***	0.05**	0.05*	0.06*	0.08***
Multiple *R*^2^	0.04	0.04	0.04	0.05	0.06	0.09	0.07	0.10	0.13	0.12	0.11	0.07	0.11

*n*_Seasons_ = 1,844–6,789. *n*_Overall_ = 18,816. Shown are standardized regression coefficients (β). Orange colors (in levels ≤ 0.04, 0.05–0.09, 0.10–0.14, and ≥ 0.15) indicate significantly positive relationships and green colors (in levels ≥ −0.04, −0.05–0.09, −0.10–0.14, and ≤ −0.15) indicate significantly negative relationships. *p < 0.05; **p < 0.01; ***p < 0.001.

Investigating the role of behavior for concerns revealed a clearer picture (RQ2b). Participants taking measures regarding hygiene such as using covers for mouth and nose were more likely to be concerned about COVID-19 in seven of eight covered time segments (*β* = 0.03–0.08, *p*s < 0.05). Similarly, isolation behavior (e.g., leaving home less frequently) was consistently significantly related to higher levels of concerns (*β* = 0.08–0.21, *p*s < 0.01). Increasing provisional measures (e.g., having food delivered more frequently) positively predicted being concerned until spring 2022 (*β* = 0.04, *p*s < 0.01), but the effect was no longer statistically significant in the four time segments after that.

In terms of RQ2c, several COVID-19-related perceptions predicted concerns. Perceived controllability of risk of infection was significantly related to lower concerns in nine of 12 time segments (*β* = −0.04–0.14, *p*s < 0.05), becoming less important from fall 2022. Respondents evaluating the mandatory use of masks as appropriate were more likely to state both higher and lower levels of concerns, depending on the time frame. There were negative associations in the beginning of the pandemic in summer and fall 2020 (*β* = −0.03, *p*s < 0.05) and positive associations in eight of 10 remaining time segments after that (*β* = 0.03–0.09, *p*s < 0.05). Alternatively, reported appropriateness of the cancelation of events did not significantly predict concerns with the exception of spring 2021 (*β* = 0.05, *p* < 0.001). Those participants feeling more informed about the virus (e.g., symptoms) were less likely to wbe concerned about COVID-19 in four of five segments (*β* = −0.06–0.09, *p*s < 0.05), while feeling being informed about societal implications (e.g., current infection numbers) was only significantly related to lower concerns in spring and winter 2022 (*β* = −0.06–0.07, *p*s < 0.01). Finally, perceived probability of infection in both private (*β* = 0.09–0.14, *p*s < 0.001) and public settings (*β* = 0.05–0.15, *p*s < 0.05) positively predicted levels of concerns about COVID-19 in all of the five investigated time segments.

## 4 Discussion

This study extends the extant literature on infectious disease risk perceptions by examining the time-dependent trajectory of concerns about the impact of COVID-19, with a particular focus on the previously understudied effect of season. The results suggest that the development of concerns is subject to an interplay of individual factors, behavioral patterns, and beliefs based on the enduring influence of key variables.

The results on overall patterns and seasonal changes in concerns (RQ1) indicated that the concerns in different domains of people’s lives were similar over the period under consideration, but with some variations. Social concerns took on a special position in comparison, which underlines the influence of the pandemic on personal relationships, as already demonstrated in other studies (4, 43). Although the differences are within the range of less than one scale point, they are meaningful in view of the sample size and the observation over a period of 3 years.

Exploring the effect of seasons, there was a higher between-seasons than within-seasons variability in concerns. Physical health concerns showing significant differences by season including higher values in fall and winter may reflect seasonal specifics in health-related behaviors (e.g., physical activity) (44) or the confluence of the flu season (45). A seasonal pattern could also be identified for mental concerns, in which the values for winter and spring were more similar than those for summer and fall. In addition to the intensified reporting of COVID-19 in the colder seasons with a particular focus on increasing infection numbers and the uncertainty associated with this (46), the compounding effect of cold weather and the influence of reduced daylight could also be explanations. For economic concerns, the pattern was similar to that of physical health concerns, with a significant effect of season. Changing perceptions of the economic stability of the country may play a role here, as well as corresponding differences in lifestyle costs (e.g., vacation in summer). Moreover, there was an effect of season on social concerns, with the pooled mean values for summer exhibiting a different pattern than those for the other seasons. The results suggest that seasonally different arrangements of social activities and the perceived intensity of interpersonal relationships may have an impact on assessments of the pandemic. Further explanations may also lie in the effect of lockdowns (17, 37) or some preventive measures being modified quickly due to ineffectiveness (e.g., closure of certain shops), as well as in the fact that seasons may differ between different years.

With regard to RQ2 on the predictors of concerns over time, the results point less to an effect of season than to consistent influencing factors. With regard to the role of individual characteristics (RQ2a), there was first a gender disparity in reported concerns about COVID-19, with females consistently expressing higher levels. This finding aligns with research on gender-based differences in health-related anxieties (47), but raises the question for future works as to which underlying factors should be investigated for a more nuanced explanation. In addition, lower education was found to be a robust predictor of concerns over the entire period. Individuals with lower education tend to profit less from health-related knowledge, social inclusion, and financial stability (48), which may result in the development of concerns. With varied patterns regarding age, occupation, and household size, vulnerabilities, work-related stressors, and the perception of more household members having the chance of spreading the disease were less important when controlled for other factors.

Investigating the role of behavioral patterns for levels of concerns (RQ2b), taking hygiene measures positively affecting concerns may reflect an increased sense of vulnerability (49). Similarly, individuals who reported having taken isolation-related measures also showed higher levels of concerns. This underlines the possible psychological burden of reduced social interactions, which can translate into further thoughts about problems in other areas of life (1, 2). Taking provisional measures was positively related to concerns as well, but the effect diminished in summer 2022 as pandemic-related disturbances in supply chains may have been publicly discussed to a lesser extent. Importantly, potential circular relationships between perceptions and behavioral patterns should be taken into account. Concern and other dimensions of risk perception (e.g., persistent thinking about an issue) (50) may also affect individuals’ ability to adapt to circumstances, influencing actions. Suggesting resilience as a potential explanatory mechanism, Morales-Vives et al. (51) found more resilient participants to be more likely to better adapt to pandemic measures. Further, season may impact this resistance to circumstances (e.g., in summer with different resting times).

Echoing the literature on perceptions of control and risk (52), perceived controllability of the risk of infection emerged as a key predictor of higher levels of concerns within the block of perceptions (RQ2c). Moreover, feeling more informed about both the virus and its toll on society positively predicting concerns emphasizes the role of believing to be accurately informed in alleviating risk perceptions (22, 53). Positive relationships of perceived probability of infection in both private and public contexts and concerns may be explained by the significance of perceived threat in shaping individual apprehensions (54). Acknowledging the ongoing discussion about the interpretation of effect sizes (55), it should be noted that these were comparatively small, calling for caution in deriving practical relevance from them.

Nonetheless, these results have implications for future risk and crisis communication. Communication should be tailored to seasonal patterns to take into account variations in concerns. The specific impact on social concerns should be acknowledged and addressing these can help to raise awareness of social networks in a prolonged crisis. Understanding disparities in concerns depending on gender and education can guide the design of specific formats. Furthermore, the results suggest a greater focus on the link between the adoption of measures and the development of concerns; individuals who engage in desirable behaviors may be more psychologically burdened even though their actions are beneficial. Finally, communicators should recognize that people’s perceptions adapt to changing circumstances, which makes it necessary to monitor the potential influence of public discussions.

## 5 Limitations and conclusion

Limitations of this study should be acknowledged. First, it is important to emphasize that the data basis for this study were serial cross-sectional data, which do not allow causal conclusions to be drawn. Although the results indicate a time course, they do not reveal changes in perceptions and behaviors in individual subjects, making it difficult to compare different years and seasons within years (including different pandemic management strategies). These variations should be confirmed in future, timely longitudinal studies.

Second, the measurements used were based on self-reports. It should be noted that reports of concerns may be subject to social desirability and, in particular, reports of past behavior may be biased by recall issues. Experience sampling may provide a solution for future studies in this context. Moreover, future research could expand by considering the everyday relevance of an issue, facilitating a more nuanced understanding beyond concern as only one dimension of risk perception (50).

Finally, the results refer to Germany, a central European country with distinct differences in seasons. This western population in the northern hemisphere may differ from others culturally and participants in other countries may perceive seasonal variations differently, calling for studies on this topic in other latitudes and caution in using the results for international communication strategies.

In conclusion, the results provide evidence for the dynamic nature of concerns in different areas of life during a pandemic and the interplay of individual, behavioral, and cognitive factors in their development. While there is considerable research on the influence of seasonality on infection rates, it also appears to have an impact on perceptions. Although future public health crises might have other causes such as different pathogens, this understanding can be a starting point to find adequate responses to emerging concerns in order to mitigate effects on people’s lives.

## Data availability statement

The raw data supporting the conclusions of this article will be made available by the authors, without undue reservation.

## Ethics statement

Ethical approval was not required for the studies involving humans because no personally identifiable information, including sensitive or confidential data, was collected. Studies did not deal with medical aspects or involve an experimental manipulation. The service provider conducting the field work fulfills the requirements of ISO 20252:2012 for market, opinion and social research, adheres to the ICC/ESOMAR ethics code for social research and data analytics, and operates by the standards established by the Working Group of German Market and Social Research Institutes (ADM). The studies were conducted in accordance with the local legislation and institutional requirements. Written informed consent for participation was not required from the participants or the participants’ legal guardians/next of kin in accordance with the national legislation and institutional requirements because they stated their consent to participate in the surveys and could opt out or skip questions at any time. Data were collected and processed in anonymized form.

## Author contributions

RJ: Conceptualization, Formal analysis, Methodology, Project administration, Writing – original draft, Writing – review & editing. DB: Formal analysis, Methodology, Writing – review & editing. G-FB: Supervision, Writing – review & editing.

## Footnotes

^1^These data have been the basis for analyses in previous publications, see (33, 34).
