# Supplementary material for: Investigating seasonal changes in factors associated with COVID-19 concerns: Results from a serial cross-sectional survey study in Germany between 2020 and 2023
Source: Front Public Health. 2024 Jul 18;12:1397283. doi: 10.3389/fpubh.2024.1397283 (PMC11291447; doi:10.3389/fpubh.2024.1397283)
Supplement: Supplementary file 1 [file Table_1.DOCX]

Supplementary Material

**Table 1**. *Division of participants by season.*

| # | Season | *n* | Waves | Gender (female) | Age (*M*, *SD*) |
| --- | --- | --- | --- | --- | --- |
| 1 | Summer 2020 | 6,157 | 11–16 | 53.6% | 57.6 (17.5) |
| 2 | Fall 2020 | 7,099 | 17–23 | 52.6% | 56.4 (17.4) |
| 3 | Winter 2020 | 6,050 | 24–29 | 51.7% | 56.9 (17.5) |
| 4 | Spring 2021 | 7,094 | 30–36 | 52.4% | 57.5 (17.9) |
| 5 | Summer 2021 | 6,046 | 37–42 | 51.9% | 55.8 (17.4) |
| 6 | Fall 2021 | 7,048 | 43–49 | 51.5% | 57.4 (17.8) |
| 7 | Winter 2021 | 6,031 | 50–55 | 52.4% | 57.6 (17.2) |
| 8 | Spring 2022 | 7,016 | 56–62 | 52.8% | 58.4 (17.7) |
| 9 | Summer 2022 | 3,010 | 63–65 | 50.3% | 58.9 (17.3) |
| 10 | Fall 2022 | 2,988 | 66–68 | 50.8% | 60.1 (16.8) |
| 11 | Winter 2022 | 2,963 | 69–71 | 50.2% | 57.3 (17.6) |
| 12 | Spring 2023 | 1,969 | 72–73 | 46.9% | 58.2 (17.4) |

*N* = 63,471.

**Table 2**. *Zero-order correlations of variables.*

|  | 1 | 2 | 3 | 4 | 5 | 6 | 7 | 8 | 9 | 10 | 11 | 12 | 13 | 14 | 15 |
| --- | --- | --- | --- | --- | --- | --- | --- | --- | --- | --- | --- | --- | --- | --- | --- |
| 1. Gender | – |  |  |  |  |  |  |  |  |  |  |  |  |  |  |
| 2. Age | .07 | – |  |  |  |  |  |  |  |  |  |  |  |  |  |
| 3. Education | –.05 | –.05 | – |  |  |  |  |  |  |  |  |  |  |  |  |
| 4. Occupation | .04 | .50 | –.16 | – |  |  |  |  |  |  |  |  |  |  |  |
| 5. Household size | –.04 | –.45 | .04 | –.26 | – |  |  |  |  |  |  |  |  |  |  |
| 6. Protective measures – hygiene | .09 | –.03 | –.04 | .01† | –.00† | – |  |  |  |  |  |  |  |  |  |
| 7. Protective measures – isolation | .05 | .08 | –.07 | .08 | –.07 | .45 | – |  |  |  |  |  |  |  |  |
| 8. Protective measures – provision | .07 | –.01† | –.01† | .01† | –.00† | .46 | .38 | – |  |  |  |  |  |  |  |
| 9. Controllability of risk | .00† | .09 | –.06 | .11 | –.09 | .01† | .02 | .02 | – |  |  |  |  |  |  |
| 10. Appropriateness – masks | –.07 | –.11 | –.03 | –.11 | .06 | –.26 | –.21 | –.18 | .02 | – |  |  |  |  |  |
| 11. Appropriateness – events | –.05 | –.09 | –.04 | –.08 | .06 | –.22 | –.23 | –.16 | .01† | .38 | – |  |  |  |  |
| 12. Feeling informed – virus | .08 | .01† | .13 | –.05 | .01† | .10 | .05 | .09 | .09 | –.09 | –.09 | – |  |  |  |
| 13. Feeling informed – society | .07 | .14 | .08 | .04 | –.05 | .13 | .09 | .12 | .11 | –.11 | –.12 | .69 | – |  |  |
| 14. Infection probability – private | .04 | –.06 | –.07 | –.02 | .10 | .09 | .10 | .09 | –.08 | –.08 | –.08 | –.01† | –.01† | – |  |
| 15. Infection probability – public | .10 | .04 | –.00† | .03 | –.03 | .27 | .27 | .21 | –.14 | –.31 | –.28 | .08 | .09 | .25 | – |
| 16. COVID-19 concerns | .09 | .02 | –.13 | .04 | –.04 | .16 | .22 | .13 | –.05 | –.04 | –.07 | –.08 | –.06 | .16 | .17 |

*N* = 63,471.
Pearson correlations based on listwise deletion.
Correlations significant at *p* < .05 with the exception of those tagged with †.
